# Accelerating next generation sequencing data analysis with system level optimizations

**DOI:** 10.1038/s41598-017-09089-1

**Published:** 2017-08-22

**Authors:** Nagarajan Kathiresan, Ramzi Temanni, Hakeem Almabrazi, Najeeb Syed, Puthen V. Jithesh, Rashid Al-Ali

**Affiliations:** Biomedical Informatics, Research Branch, Sidra Medical and Research Center, Post Box No. 26999 Doha, Qatar

## Abstract

Next generation sequencing (NGS) data analysis is highly compute intensive. In-memory computing, vectorization, bulk data transfer, CPU frequency scaling are some of the hardware features in the modern computing architectures. To get the best execution time and utilize these hardware features, it is necessary to tune the system level parameters before running the application. We studied the GATK-HaplotypeCaller which is part of common NGS workflows, that consume more than 43% of the total execution time. Multiple GATK 3.x versions were benchmarked and the execution time of HaplotypeCaller was optimized by various system level parameters which included: (i) tuning the parallel garbage collection and kernel shared memory to simulate in-memory computing, (ii) architecture-specific tuning in the PairHMM library for vectorization, (iii) including Java 1.8 features through GATK source code compilation and building a runtime environment for parallel sorting and bulk data transfer (iv) the default ’on-demand’ mode of CPU frequency is over-clocked by using ’performance-mode’ to accelerate the Java multi-threads. As a result, the HaplotypeCaller execution time was reduced by 82.66% in GATK 3.3 and 42.61% in GATK 3.7. Overall, the execution time of NGS pipeline was reduced to 70.60% and 34.14% for GATK 3.3 and GATK 3.7 respectively.

## Introduction

The impact of next generation sequencing (NGS) technologies in revolutionizing the biological and clinical sciences has been unprecedented^1, 2^. Such projects are generating large volumes of data that need careful analysis to identify the variants that differ individually or at a population scale. However, the analysis of high throughput genome sequencing data is computationally demanding involving multiple steps with several sequential operations using open source or proprietary software tools. It is imperative to use High Performance Computing (HPC) systems to improve the performance and speedup of the workflow execution for timely and fast analysis of the data^3, 4^. It is also important to increase resource utilization efficiency and improve the throughput when analyzing data from multiple samples, which can be achieved through parallel execution^5^. Furthermore, automation of the workflow using schedulers provide robustness and ease of operations^6^. The application tools that are from part of such a workflow generally have diverse characteristics such as being compute, memory or Input/Output (I/O) intensive. When implementing a workflow system, these characteristics need to be addressed differently. For example, tools that are compute intensive may be accelerated through the use of high frequency processors; in-memory operations help in managing memory intensive tools; and a parallel file system may help in the case of I/O intensive tools^7, 8^. Such heterogeneity makes it hard to implement optimal workflows for large scale processing of huge volumes of data.

The variant detection workflows are wildly used by the research community. Burrows-Wheeler Aligner (BWA)^9^ is developed by Wellcome Trust Sanger Institute and the Genome Analysis ToolKit (GATK)^10^ is developed by the Broad institutes are among the most used tools in the NGS workflow^11^. There are two major steps in these workflows: genome alignment or mapping and variant discovery^10^. Examples of publicly available tools that are commonly used to achieve these goals in workflows construction following the recommendations include BWA and GATK respectively. There are several ways to improve the performance of these tools. For example, the execution time of genome alignment using BWA can be improved by parallelization that includes: (a) thread-parallelization by using multi-threads^12^, (b) data-parallelization by splitting the input into distinct chunks or intermediate data followed by processing the chunks one-by-one within or across the node^13^, and (c) data-parallel with concurrent execution by splitting the data into disjoint chunks then, distributing the intermediate data (chunks) across multiple CPUs and executing multi-threads within a CPU on a cluster environment^14^. The modern HPC architectures contains multi-cores within a CPU and multi-CPUs are available within a single node. Due to this modern architecture features, (i) data-parallelization can be implemented across multi-CPUs, and (ii) thread-parallelization can be done within a CPU, then these two implementations (i and ii) will be repeated concurrently across all the CPUs within node. Thus the data-parallelization, thread-parallelization and concurrent execution steps are possible within a node.

The genome alignment requires large amount of memory that are addressed by modern HPC systems. Non-Uniform Memory Access (NUMA) based multi-CPU is the features of modern HPC architecture and more than 2 Terabyte of main memory can be possible within a single node. To improve the execution time, in-memory computation and data-parallelization are prerequisite for genome alignment^15^. In addition to data-parallelization (e.g. distribution of independent chunks of data across the CPUs), concurrent parallelization (e.g. multi-threading) is implemented in multi-core CPUs of modern HPC systems^5, 14^. These types of BWA optimizations are done in our earlier paper^3, 14^ in the traditional HPC system. These implementations simulate in-memory computing concept, which may bring better performance benefit but fails in resource utilization^14^. To address this issue, we proposed optimization of data intensive computing model by using optimal number of multi-threads for every sample execution and processing multiple samples within a node in an empirically parallel manner^3^. Thereby, the execution time will be improved, multiple-samples are processed at the same time and optimal utilization of the computing resources are achieved^3, 5, 14^.

Most of the variant discovery algorithms fail to scale-up on multi-core HPC systems and this results in multi-threading overhead, poor scalability and underutilization of HPC resources^16^. To address these challenges, data-parallelization and pipeline parallel execution models are implemented in ref. 5 and an optimized data partition method was used in refs 3 and 15. Partitioning the data into multiple segments for data-parallelization and distributing the segments across different CPUs for pipeline parallel execution are challenging tasks. Additionally, merging of all the partial output files into a resultant file in the same order of file split is more important (when the mapped files are sorted) to ensure the correctness of the results. The optimized data partition uses granularity-based and individual-based partitioning methods. Generation of optimal number of small chunks for the best performance and resource utilization has been a matter of debate^3, 6, 15^. The selection of optimal number of chunks may vary across the clusters. Once the optimal number is derived, the sample data need to be split into multiple chunks for the genome analysis with the reference genome. The cache fusion was used to improve the performance of genome alignment, choke elimination was used to eliminate the waiting time in the workflow and the framework of merged portion algorithm was invoked for better performance and optimal resource utilization in an optimized data portion model^17^.

## Methodology and Benchmarking Environment

### Systems & Software environment

Intel Sandy Bridge system that has four E5-4650 CPUs @ 2.7 GHz, 8 physical cores per CPU, totaling 32 cores used for benchmarking. This system has NUMA architecture with 256 GB DDR3 memory and Red Hat Enterprise Linux (RHEL, version 6.4) is an operating system. We used different versions of Java (1.7 and 1.8) and GNU Compiler 5.2.0 for our benchmarking experiment.

### Dataset used for benchmarking

1. **Reference genome:** Human genome reference build GRCh37^18^, gold standard INDELs-Insertion and Deletions (Mills_and_1000G_gold_standard.indels.vcf.gz)^19^ and variant database build 138 (dbSNP, dbsnp_138.vcf.gz)^20^ are the set of genome reference files. Additionally, the TruSeq universal adapter^21^ is used for trimming the paired-end sequencing.
2. **Input dataset:**

- **GCAT genome data:** Genome Comparison & Analytics Testing (GCAT)^22, 23^ provides a benchmarking datasets. We used 10 different genome datasets (gcat set 037–039, 041–046 and 049) for benchmarking which covers the sequence length of 100 base pairs and 150 base pairs, for both paired end large-indel data set. These data set are used to optimize the HPC system tuning parameters that includes (i) performance tuning in the Java parallel garbage collection, (ii) performance effect of CPU frequency scaling and (iii) kernel shared memory tuning. We used this GCAT data to tune our HPC system because of (a) standard and universal dataset for benchmarking (b) the execution time is very small (e.g. 1–3 hours) and (c) dataset is in few MB size. Hence, we can more frequently modify the various HPC system parameters for better execution time before running the larger genome data (e.g. platinum genome) in a production environment.
- **Platinum genome project data**^24, 25^: To compare the real-time performance improvement and the optimization benefits, we used the platinum genome data from Illumina HiSeq X10 sequencers. Moreover, these platinum genome data set are relatively similar characterization to the above said GCAT data, but the volume of data are huge (65–110 GB, pair-ended and large indels) and the execution time are in days (1–6 days). We used this data set to compare the execution time of (i) baseline performance and (ii) architecture aware tuning of GATK results. We used 3 different data set from Illumina Whole-Genome sequencing project^26^.

3. **Data coverage:** The mean/median coverage of (a) GCAT genome data has 10 different mean/median values (13.66, 21.34, 22.19, 25.34, 22,21, 25.18, 23.43, 23.39, 24.35 and 24.26) and (b) platinum genome are 3 different mean/median values (51.61, 49.753 and 54.35) for the better benchmarking results. The mean/median coverage are calculated using Picard tools^27^ and the example script are available in Supplemental Data IV.

### Benchmarking methodology

#### HaplotypeCaller for performance benchmarking

Amdahl’s law recommends to optimize the most time consuming part of the code in the application^28^. Additionally, the overall speedup of the application will be improved when the most time-consuming part of the code is optimized. Therefore, the optimization strategy should be a part of the code where the work will pay-off with the biggest gain in the performance. As per our NGS workflow, the HaplotypeCaller is consuming more than 43% of the total execution time and hence we selected the HaplotypeCaller for benchmark evaluation and optimization.

#### Performance evaluation metrics

The performance evaluation is based on the application execution time. In a parallel and distributed computing environment, the workload is distributed across the CPUs and the total CPU time is more than the “real-time”. Hence, the “real-time” (e.g. UNIX command time −p) is used to measure the execution time.

### List of workflow modules and different type of parallelization

The BWA^9^ and GATK^10^ are the two modules that are used in our NGS workflow. The BWA is a popular genome alignment tool and it consists of three algorithms: BWA-backtrack, BWA-SW and BWA-MEM. These algorithms supports (i) thread parallelization (ii) data-parallelization and (iii) data-parallel with concurrent execution^3, 14, 29^. The GATK supports two type of parallelization: (i) data parallelization and (ii) thread parallelization. The ‘data parallelization’ is controlled by number of “data threads” per process by using −nt = 〈number of data threads〉 at runtime. The ‘thread parallelization’ is controlled by the number of “CPU threads” that are allocated per data threads. This thread-parallelization is controlled by using −nct = 〈number of CPU threads〉 option during the runtime. To understand the best parallelization performance, we tested the BWA and GATK with varying number of cores from 1 to N and used the optimal number of cores for this performance case studies. The summary of various parallelization strategies are summarized in Supplemental Data I.

### Workflow implementation for genome mapping

The preliminary step is to mapping the genome sequencing data in the form of FASTQ files into Sequence Alignment/Map (SAM) format or BAM (Binary version of SAM) format, marking the duplicates, merging and sorting the genome data and the genome variant discovery^30^. For the genome alignment, we used BWAKIT^31^, which is an open-source tool that includes the pre-compiled ×86_64 binaries of Seqtk^32^, SAMtools^33^, Trimadap^34^, BWA-MEM^35^ and Samblaster^36^. Seqtk is a fast and lightweight toolkit for processing sequences in the FASTA/ FASTQ format. Trimadap is used for trimming the adapter sequence from the FASTQ files. The latest version of BWA algorithm is BWA-MEM that provides fast and accurate alignment of genome sequence, supporting long-query and split-alignment^4, 13^. Samblaster helps in marking duplicates as removing duplicates is important to mitigate the effects of polymerase chain reaction (PCR) amplification, and reducing the number of reads to be processed during variant discovery. Sambamba is optionally used for indexing^37^.

#### Some modification in the GATK best practices workflow

We used the TruSeq universal adapter^21, 32^ for trimming the paired-end sequencing, which is part of the BWAKIT package. We preferred to use Samblaster^36^ (stand-alone tools) for duplicate marking because it performs significantly better than Picard^27^. Additionally, the execution time and memory usage of Samblaster is better than Picard. Moreover, the results obtained from Samblaster is nearly identical results of Picard^36^.

As a summary, BWA 0.7.15, BWAKIT 0.7.15, SAMtools 1.3, seqtk 1.0, samblaster 0.1.20, V8: 3.16.14 and K8: 0.2.1 are the version history, which we used during our benchmarking. The example BWAKIT pipeline is available in Supplemental Data VI.

### Workflow implementation for variant discovery

For the variant discovery stage, we focused on the identification of Single Nucleotide Variants (SNV) and small INDELs using GATK. The analysis-ready BAM files are the input for the variant discovery phase. We have used the following modules from GATK for our variant discovery.

1. **Realigner TargetCreator:** The analysis-ready BAM files are locally re-aligned using this step. The realigned intervals are produced after the successful execution. The misalignment to the reference genome are identified using the realigned interval which are very useful in SNPs detection.
2. **IndelRealigner:** It perform the local realignment of the genome reads within small INDELs and its produces the realigned BAM files. Note that, starting from GATK 4.0, this Indel realignment step will be no longer part of the NGS workflow.
3. **BaseRecalibrator:** The base recalibration table will be generated to minimize the systematic errors.
4. **PrintReads:** A sequence of read data is written by the ReadWalker algorithm. This algorithm reads a recalibration table and the realigned BAM files. It produces a realigned, re-calibrated BAM files, which includes filtering, merging and subsetting of the genome data for the meaningful genome analysis.
5. **HaplotypeCaller:** The ActiveRegionWalker algorithm is used for local re-assembly of haplotypes and it is possible to call the SNPs and small INDELs simultaneously. The variant validation is an important stage in this genome analysis and it’s possible to reassemble the reads when any variants are detected.
6. **Variant Recalibrator:** The LocusWalker algorithm is used to build a recalibration model. It is useful for variant quality analysis and filtering purposes.
7. **Analyze Co-variates:** The variant analysis plots are created to visualize the base recalibration results.

**Figure 1:**
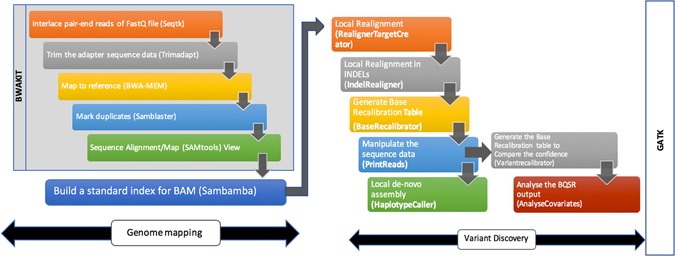
Next Generation Sequencing analysis workflow for the discovery of new functional variants.

We developed and automated the genome variant discovery pipeline to process multiple genomic samples in our HPC environment. The automation of the workflow is developed based on the job dependency conditions. The example workflow scripts are available in https://github.com/sidratools/Genome-workflow and the summary of the NGS workflow is shown in Fig. 1.

Over the last three years, there are two major versions of GATK (GATK 3.x and GATK 4.0) are released. The Java 1.7 and Java 1.8 are used to develop GATK 3.5 (and older versions) and GATK 3.6 (and later versions) respectively. To understand the best execution time of GATK, the two different implementations (Java 1.7 and Java 1.8) of the GATK 3.x are used to build the NGS workflow and benchmarked with various system level optimizations. Since the GATK 4.0 implementation was completely different from GATK 3.x, we are not compared the execution time of GATK 3.x to GATK 4.0. Moreover, the GATK 4.0 is supported for the Spark architecture which is different from the traditional HPC systems.

The GATK 3.5 and the older versions are not developed with Java 1.8. But, the GATK 3.6 and GATK 3.7 are developed using Java 1.8. To understand the performance benefit of Java 1.8, we compiled the GATK 3.5 and other older versions using Java 1.8 and benchmarked with JDK 1.8 runtime environment. We benchmarked GATK 3.3, 3.4 and 3.5 versions with Java 1.8 support. For every version of these GATK, we used two different executables: (i) pre-compiled jar file (referred as “GATK 3.x_jar”) (ii) source code compiled GATK executable (referred as “GATK 3.x_src”). The GATK PrintReads and HaplotypeCaller execution time of these various versions are summarized in Supplemental Data II. We selected the source code compiled GATK 3.3 executable for benchmarking this workflow implementation because of it is more stable, bug free and 23% better performance than other versions. The execution time of GATK 3.3 version is compared to the latest GATK 3.7 version for this system level optimization of NGS workflow.

## Results

### Baseline performance

To provide a baseline results for the system level optimization, we listed the various job steps for the NGS workflow, its’ associated software tools or modules, application parameters, input and output file formats, job dependency conditions, recommended number of cores and the percentage of execution time (for each of the steps) are given in Table 1. These execution timings are obtained from various performance engineering case studies that are conducted previously^3, 5, 8, 14^. The PrintReads and HaplotypeCaller consume 25% and 43% of the execution time respectively and totaling >65% of time in the NGS workflow.

**Table 1:** Computational steps, dependency conditions and their execution time in the NGS workflow.

Step ID	Job step name	Application name	Application module	Input file name	Application parameters	Output file name	Recommended no. of cores	Job dependency condition	% of execution time
S1	Map to Reference	BWA KIT	Seqtk, trimadap, SamTools, bwa mem, samblaster	*.fastq.gz	Default	*.bam	N/M	—	6.5%
S2	Build a standard BAM INDEX	sambamba	Index	*.bam	Default	*.bam.bai	1	S1	0.5%
S3	Realigner TargetCreator	GATK	Target creator	*.aln.bam	−*T* RealignerTargetCreator, −*R* hs37d5.fa, −*known* Mills_and_1000G_ gold_standard.indels.vcf.gz,	*.realigner. intervals	4 or 8	S2	3%
S4	Indel Realigner	GATK	INDEL	*aln.bam, *.realigner. intervals	−*T* IndelRealigner, −*R* hs37d5.fa, −*known* Mills_and_1000G _gold_standard.indels.vcf.gz, −*knownIntervals*	*.realigned. bam	1	S3	2%
S5	Base Recalibrator	GATK	Base Recalibration	*.realigned. bam	−*T* BaseRecalibrator, −*R* hs37d5.fa, −*k*nownSites dbsnp_138.vcf.gz	*.recal.table	N/M	S4	13%
S6	Print Reads	GATK	Analyse the Reads	*.realigned. bam, *. recal.table	−*T* PrintReads, −*R* hs37d5.fa, −*BQSR*	*.realigned. recal.bam	2 or 4	S5	25%
S7	Haplotype Caller	GATK	Haplotype	*.realigned. recal.bam	−*T* HaplotypeCaller, −*R* hs37d5.fa, −*pairHMM* VECTOR_LOGLESS_CACHING, − −emitRef Confidence GVCF, − −variant _index_type LINEAR, − −*variant*_index_parameter 128000, − −dbsnp Mills_and_1000G_ gold_standard.indels.vcf.gz	*.raw.snps. indels.g.vcf	4 or 8	S6	43%
S8	Variant Recalibrator	GATK	Variant recalibration	*.realigned. bam, *.recal.table	−T BaseRecalibrator, −R hs37d5.fa, −known Mills_and_1000G_ gold_standard.indels.vcf.gz, −BQSR	*.after_recal. table	N	S5	6%
S9	Analyze Covariates	GATK	Analyse the variant	*.recal.table, *.after_ recal. table	−T AnalyzeCovariates −before −after	*.recal_plots. pdf	1	S8	1%

Where, N is the total number of cores and M is the number of CPUs.

### Performance tuning in Java and garbage collection

#### Execution time of GATK 3.3

We benchmarked the execution time of GATK 3.3 during the changes of various parameters which includes, Java version, garbage collection, heap and stack size of Java virtual machine that are summarized as follows:

1. **Java stack size:** By default, the Java stack size (e.g. −Xss10m) is same as that of the operating system stack size (i.e, 10 Mb). With the increase of stack size, we did not observe any improvement in the GATK execution time (data not shown).
2. **Java heap size:** We observed there is no changes in the GATK execution time when the heap memory is reduced <50% of the main memory available in the benchmarking system. Further, we observed the GATK execution was failed for the larger genome data (e.g.: platinum genome) when the heap memory is <50% of the main memory size. Hence, we recommended to use at-least 50% of the main memory for the heap size.
3. **Parallel garbage collection:** In a multi-CPU system, the parallel garbage collection (−XX: +UseParallelGC and −XX:ParallelGCThreads = ‘no. of CPU threads’) gives the best execution time. Further, it is recommended to increase upto maximum number of CPU threads (as per the benchmarking system architecture) when the number of cores are large. We observed the GATK execution time was improved upto 28%, when the parallel garbage collection uses 32 CPU threads.
4. **CMS garbage collection:** Asynchronous operations is the feature of CMS garbage collection in the modern multi-core architecture. All the threads can independently progressed when this CMS garbage collection is invoked by using −XX: +CMSParallelRemarkEnabled. It is referred as low latency garbage collection and mostly used in modern HPC architectures. We observed, the execution time between CMS garbage collection and parallel garbage collection are comparable and gives the similar execution time.
5. **Out of memory error in Java 1.7:** The Permanent Generation (PermGen) space is used to store the Java class objects. The PermGen will be running out of the space when more Java class objects are loaded. As a result, either the application may not start or it will fail at java.lang.OutOfMemoryError. To eliminate these errors, the PermGen space can be re-defined or increased using the option −XX:MaxPermSize = ‘size of PermGen’. Sometimes, the preallocated PermGen space may not be sufficient (e.g. −XX:MaxPermSize = 512m) due to more Java class objects will be loaded at runtime or memory leak due to the poor coding of the applications. We observed the OutOfMemoryError during the execution of the HaplotypeCaller in the standard gCVF mode (−variant_index_type = LINEAR and variant_index_parameter = 128000). Moreover, the HaplotypeCaller in the standard gVCF mode consume huge memory, additional class objects files and third party libraries (e.g. PairHMM libraries) are loaded at runtime. As a result, when the PermGen space is limited or exceeded the preallocated size and the HaplotypeCaller will keep crashing with java.lang.OutOfMemoryError.
6. **Java 1.8 features:** Java 1.8 is recommended to eliminate the PermGen limitations and runtime issues (java.lang.OutOfMemoryError). The PermGen space was removed in JDK 1.8 and the virtual machine size was very small (small VM). As a result, OutOfMemoryError is eliminated in Java 1.8. Additionally, the parallel array sorting and bulk data transfer operations are important features which are more beneficial for our NGS workflow. Java 1.8 support was included from GATK 3.6 version and not officially supported until GATK 3.5 or older versions. We used Java 1.8 for compilation of GATK 3.3, 3.4 and 3.5 versions and JDK 1.8 is used as a runtime environment. Moreover, all our benchmarking experiments are successfully run with Java 1.8, eliminated OutOfMemoryError for the HaplotypeCaller in the standard gVCF mode and demonstrated the best performance.
7. **Java temporary I/O file directory:** The temporary I/O file directory can be a local file system (/tmp) or a parallel file system (/gpfs) that can be modified at runtime using −Djava.io.tmpdir = ‘directory location’. When the local file system is used, all the Java threads writes a temporary files into a local disk within a computing system. Alternatively, all the computing system within a cluster can access a parallel file system and all the Java threads can write a temporary files into a parallel file system directories simultaneously. The GATK execution time is −21% poor and 5% better for Java 1.7 and Java 1.8 respectively, when a parallel file system (/gpfs) is used as a temporary I/O file directory. The performance penalty for Java 1.7 is more when the Java I/O temporary directory is a parallel file system (/gpfs) because of the HaplotypeCaller generate the intermediate files that are <512 KB which is much lesser than our parallel file system block size (i.e., the disks are formatted with 512 MB block size). The parallel file system is trying to occupy complete 512 MB block size while wring the temporary file and it’s creates more overhead when writing a intermediate files. When the Java 1.8 is used without temporary I/O file directory, the GATK execution time was improved >52% due to Java 1.8 caching features. We observed the same performance behavior for the local file system too.

#### Execution time of GATK 3.7

The latest version of GATK 3.7 execution time is compared with the following benchmarking experiments: (i) pre-compiled GATK 3.7 jar file and (ii) the GATK 3.7 source code compiled executable are run with parallel garbage collection with 32 CPU threads. Since the development of GATK 3.7 uses Java 1.8 features, the heap memory configuration and Java temporary I/O directory are not included for our benchmarking experiment.

All the GATK 3.3 and GATK 3.7 performance results using Java, parallel garbage collection, Java temporary I/O directory execution timings are summarized in Table 2

**Table 2:** Performance effect of Java, parallel garbage collection and temporary I/O directory tuning parameters.

GATK version	Java version	Parallel GC option	Java temporary I/O directory	HaplotypeCaller execution time (in Hours)
GATK3.3_src	Java 1.7	N/A	/tmp	5.26
	Java 1.7	Parallel GC = 32	/tmp	3.81
	Java 1.7	Parallel GC = 32	/gpfs	5.03
GATK3.3_src	Java 1.8	Parallel GC = 32	/gpfs	4.78
	Java 1.8	Parallel GC = 32	—	2.51
	Java 1.8	CMS GC	—	2.52
GATK 3.7_jar	Java 1.8	Parallel GC = 32	—	1.17
GATK 3.7_src	Java 1.8	Parallel GC = 32	—	1.14

.

### Performance effect of CPU frequency scaling

The dynamic frequency scaling is used to modify the CPU frequency ‘on-the-fly’ without shutdown or rebooting the system^38^. We performed couple of CPU frequency tuning benchmarks based on in-built CPU governor readily available in the Linux kernel such as ‘performance mode’ and ‘on-demand mode’. First, all the CPU frequencies from ‘on-demand mode’ (default) is modified into ‘performance mode’. To measure the GATK execution time, an experiment was conducted in 4 socket CPU (E5-4650) running at 2.7 GHz and the theoretical power consumption of every CPU is 130 W power. The GATK 3.3 and GATK 3.7 of HaplotypeCaller with parallel garbage collection and 32 CPU threads are used for this benchmarking. When the CPU frequency is enabled as ‘performance mode’, all the 4 sockets consumed 520 W (4 × 130 W) power because of all the cores are running (always) at 2.7 GHz. We observed, more than 48% of the execution time was improved in GATK 3.3 and GATK 3.7 version when the CPU frequency is modified as a “performance mode”. The performance drop during the ‘on-demand’ mode is due to the Java threads are not always active and resulting, the CPU frequency is switching between “power-saving” or “on-demand” mode (CPU run at 1.8 GHz) to a “performance mode” (CPU run at 2.7 GHz). This benchmark experiment recommend us to enable the CPUs on “performance mode” always. This recommendation is common for any Java based applications and especially for this GATK workload. We used Intel Gadget tool^39^ to measure the power consumption. We provided the example script to set all the CPUs in the performance mode and it’s available in https://github.com/sidratools/Genome-workflow. The summary of the HaplotypeCaller execution time and electrical power consumption are shown in Table 3.

**Table 3:** Performance effect of CPU frequency scaling in the HaplotypeCaller.

GATK version	Type of CPU frequency scaling	HaplotypeCaller execution time (in Hours)	Power (in KWh)
GATK 3.3_src	Performance	2.51	1.34
	On-demand	3.72	0.47
GATK 3.7_src	Performance	1.14	0.59
	On-demand	1.49	0.19

### Performance tuning in kernel shared memory

We used the HaplotypeCaller for the kernel shared memory benchmarking because of (i) the execution time of HaplotypeCaller is more than other steps in the NGS workflow and (ii) the vectorization and advance vectorization libraries are taking advantages of kernel shared memory communication. Furthermore, most of the Bioinformatics applications are multi-threaded, executed within a single computing system, share a main memory across all the multi-threaded and a data transfer can take advantages of shared memory communication. To get the best execution time, it is necessary to tune the kernel shared memory, network read/write buffer size and read/write memory parameters of TCP/IP communication. The default kernel shared memory size will be very small (normally ≤4 GB) depending on the computing system architecture and the operating system versions. We increased the kernel shared memory from 4 GB (i.e., default size for our benchmarking system) to 128 GB (i.e., upto 50% of main memory). Also increased read/write memory buffer size of TCP/IP communication^40^ to achieve the maximum performance benefit of Java 1.8 caching features. The following kernel shared memory parameters are used to get the best execution time of GATK-HaplotypeCaller^40, 41^:

$$\begin{array}{l}\mathrm{kernel}\mathrm{.shmmax}=137438953472\\ \mathrm{net}\mathrm{.core}\mathrm{.wmem}\_\,\max \,=16777216\\ \mathrm{net}\mathrm{.core}\mathrm{.rmem}\_\,\max \,=16777216\\ \mathrm{net}\mathrm{.core}\mathrm{.wmem}\_\mathrm{default}=16777216\\ \mathrm{net}\mathrm{.core}\mathrm{.rmem}\_\mathrm{default}=16777216\\ \mathrm{net}\mathrm{.ipv4}\mathrm{.tcp}\_\mathrm{mem}=16777216\,16777216\,16777216\\ \mathrm{net}\mathrm{.ipv4}\mathrm{.tcp}\_\mathrm{wmem}=4096\,87380\,16777216\\ \begin{array}{c}\mathrm{net}\mathrm{.ipv4}\mathrm{.tcp}\_\mathrm{rmem}=4096\,87380\,16777216\\ \mathrm{net}\mathrm{.ipv4}\mathrm{.tcp}\_\mathrm{low}\_\mathrm{latency}=1\end{array}\end{array}$$

To demonstrate the performance impact of kernel shared memory with Java 1.8 features, the GATK 3.3 and GATK 3.7 versions are used for the following benchmark test: (a) The kernel shared memory is modified into 4 GB, 8 GB, 64 GB and 128 GB sizes and (b) for every kernel shared memory modifications, the HaplotypeCaller executed with 32 CPU threads (−nct = 32) and 32 parallel garbage collection threads. We observed significant performance improvement (>74%) for the GATK 3.3 and 22% improvement for the GATK 3.7. The summary of results are shown in Table 4.

**Table 4:** Performance effect of kernel shared memory.

Kernel shared memory size	HaplotypeCaller execution time for GATK 3.3 (in Hours)	HaplotypeCaller execution time for GATK 3.7 (in Hours)
4 GB	4.36	1.39
8 GB	3.01	1.39
64 GB	2.84	1.18
128 GB	2.51	1.14

### Architecture-specific tuning in the GATK-HaplotypeCaller

The HaplotypeCaller uses PairHMM kernel implementation for better execution time on the modern HPC architecture. The GATK source code supports VectorPairHMM implementations to take advantage of modern HPC vectorization and advance vectorization features. We compiled the GATK 3.3 and GATK 3.7 source-codes using apache Maven^42, 43^ with architecture aware PairHMM implementations. We used (a) vectorization and (b) advanced vectorization compiler flags to generate architecture aware HaplotypeCaller executable.

#### PairHMM implementation in GATK 3.3

The best optimization includes parallel garbage collection, Java 1.8 features, Java temporary I/O tunings, kernel shared memory parameters, CPU frequency scaling and parallelization of GATK using CPU threads are added into the GATK 3.3 runtime. The GATK 3.3 unoptimized pre-compiled jar file with CPU threads = 32 is compared to the optimized GATK 3.3 executable with the following 6 case studies: (i) Best optimization with CPU threads = 32, (ii) Best optimization with CPU threads = 8, (iii) Best optimization with vectorization instructions using CPU threads = 8, (iv) Best optimization with vectorization instructions using CPU threads = 32, (v) Best optimization with advance vectorization instructions using CPU threads = 8 and (vi) Best optimization with advance vectorization instructions using CPU threads = 32.

**Figure 2:**
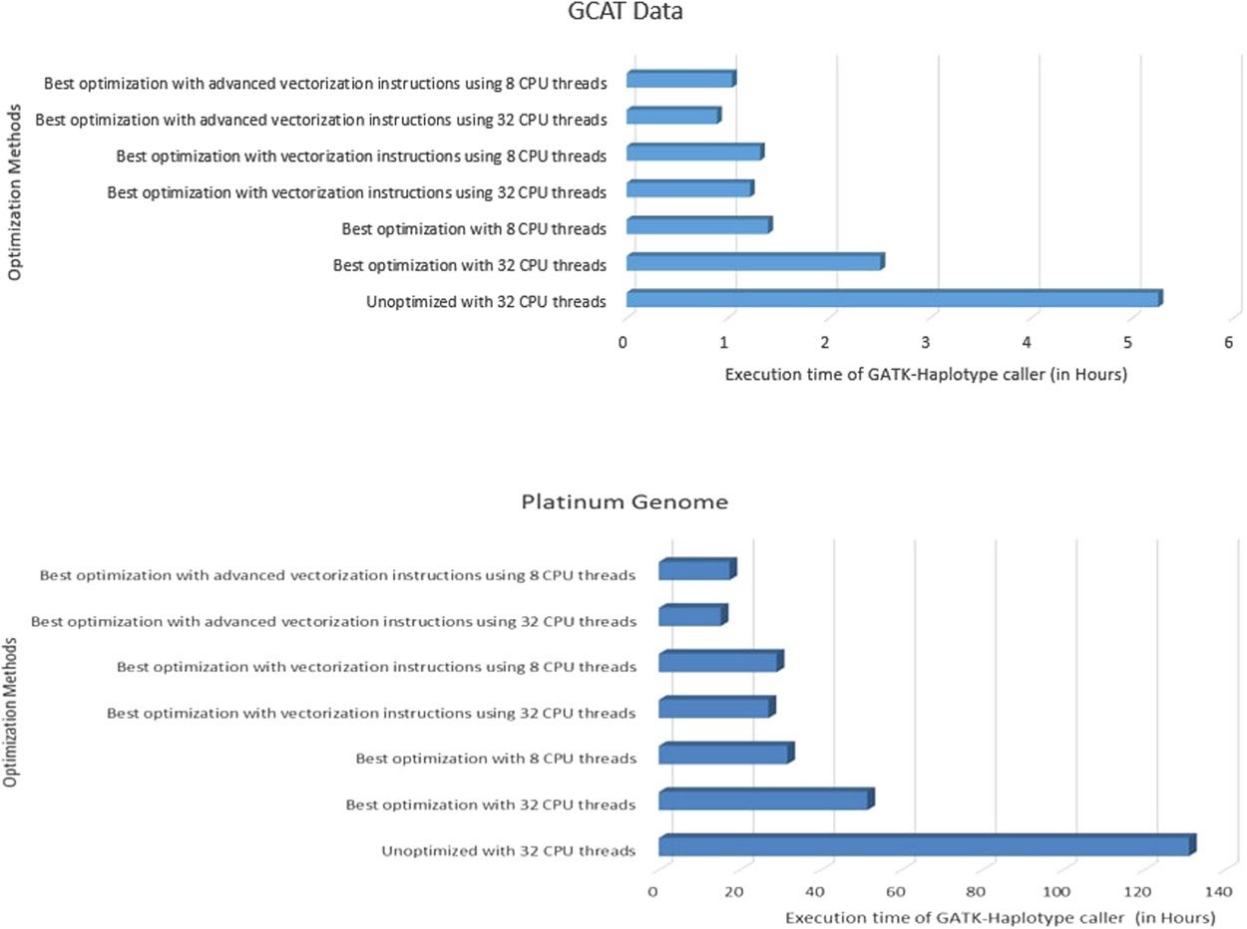
Performance impact of PairHMM vectorization in the GATK 3.3 HaplotypeCaller using architecture aware implementation.

The PairHMM implementations using vectorization and advanced vectorization of HaplotypeCaller execution bring the performance benefit of 8% and 17% respectively for CPU threads = 8. The advance vectorization brings 36% better performance for larger number of cores (e.g. CPU threads = 32) and this is 180% better execution time when compared (using similar number of CPU threads) to the best optimization results. The complete summary of results are shown in Fig. 2.

To study the performance impact of GATK execution time for the PairHMM implementation, the HaplotypeCaller was benchmarked with advanced vectorization. We used 32 CPU threads, 0 to 128 GB heap memory and GCAT data for this benchmarking. We used 4 different benchmarking experiments that are summarized in Table 5. As a result, the PairHMM library with heap memory configuration is required to get the reduced HaplotypeCaller execution time.

**Table 5:** Performance impact of PairHMM library with heap memory implementation in the GATK 3.3 HaplotypeCaller.

Benchmarking case study name	PairHMM library	Heap memory prerequisite	Committed heap memory	CPU utilization	HaplotypeCaller execution time (in hours)
Without PairHMM and No heap memory	No	0 GB	14.3 GB	upto 40%	2.51
Without PairHMM and with heap memory	No	128 GB	48.5 GB	upto 73%	2.34
With PairHMM and No heap memory	Yes	0 GB	12 GB	upto 58%	1.18
With PairHMM and with heap memory	Yes	128 GB	50.3 GB	upto 72%	0.912

#### PairHMM library implementation in GATK 3.7

To understand the performance improvement in the GATK 3.7 version, the architecture aware PairHMM library is enabled in the HaplotypeCaller and repeated the experiment using GCAT and platinum genome data. All the experiment results are summarized in Table 6. The vectorization and advance vectorization binaries gives the similar results. The PairHMM library implementation in the HaplotypeCaller with CPU threads = 8 gives the best GATK 3.7 execution time, which is 26% and 74% better than without PairHMM impementation for GCAT and platinum genome data respectively. We observed that, the CPU threads = 32 brings scalability limitations and hence we benchmarked with CPU threads = 8 for this GATK 3.7 version. Further, we observed that the optimized HaplotypeCaller execution time in the GATK 3.7 version is slightly better (1.7% reduced execution time) than Java 1.8 implementation of GATK 3.3.

**Table 6:** Performance effect of PairHMM library implementation in GATK 3.7 HaplotypeCaller.

PairHMM library	Execution time of HaplotypeCaller using GCAT genome data (in Hours)	Execution time of HaplotypeCaller using Platinum genome data (in Hours)
No PairHMM library	1.14	30.43
VectorPairHMM library	0.902	17.46

Based on all the above case studies, we provided the set of recommended values for the generic HPC architecture in Table 7 and this may be useful for basic tuning in any one of the computing system.

**Table 7:** General recommendation for the generic HPC system.

Parameter Name	Recommended value	Expected % performance improvement (in %	Remarks
Java heap size	upto 50% of main memory	N/A	GATK execution may fail due to insufficient memory when the heap size is small
Parallel garbage collection	$\mathrm{ParallelGC}$ and $\mathrm{ParallelGCThreads}$ = ‘$\mathrm{Total}\mathrm{number}\mathrm{of}\mathrm{cores}$’	upto 28%	N/A
CMS garbage collection	${\mathtt{CMSParallelRemarkEnabled}}$	upto 28%	Useful in modern HPC architecture
Java 1.8	N/A	upto 52%	Don’t use java.io.tmpdir
CPU frequency scaling	Performance mode	upto 45%	By default, modern HPC architecture uses on-demand mode
Kernel shared memory	upto 50% of main memory	upto 48%	N/A
PairHmm library with heap memory	$\mathrm{java}\mathrm{.library}\mathrm{.path}$ = ‘$\mathrm{VectorPairHMM}\mathrm{library}\mathrm{path}$’	upto 145%	Use architecture specific libraries for GATK HaplotypeCaller

## Discussion

We observed that the execution time of GATK improved (more than 50% for GATK 3.3 and slight improvement for GATK 3.7) during various tuning in parallel garbage collection. We also noticed that GATK 3.3 failed due to insufficient memory unless the heap memory is increased from default value. Since the GATK 3.7 has Java 1.8 features, we are not observed any GATK failure due to insufficient memory. Therefore, the parallel garbage collection for GATK 3.3 is an important for automatically freeing objects that are no longer referenced by the Java program. To get the best performance benefit of parallel garbage collection, the heap memory size may be increased or filled to a certain level, which is possible through the Java command line. The memory request can be invoked by using −Xms, (e.g −Xms32g) and the maximum level can be set by using −Xmx, (e.g. −Xmx128g). Note that the error “Not enough memory to run GATK” is common at GATK run-time which can be solved by increasing the Java heap memory size (upto 50% of the main memory). For example: When the HPC system has 256 GB of main memory, the heap memory can be increased upto 50% (−Xmx128g, −Xms128g).

Most of the modern Linux operating systems support dynamic frequency scaling to save electrical power and thermal energy. By default, “on-demand” mode is active in the modern operating system^38^. We observed, more than 48% of the execution time was dropped (both GATK versions) when the CPUs are running at “on-demand” mode. To get the best performance, we recommend to set the CPUs in “performance mode” for our NGS workflows.

The HaplotypeCaller uses PairHMM library for better performance. The GATK source code supports VectorPairHMM implementation and the source code is available in GATK distribution directory “public/VectorPairHMM/”. To optimize the PairHMM library, the architecture specific compiler flags (e.g. −march = core-avx2, −xAVX, −mAVX, −xAVX2, −msse4.1, −march = core-avx, −march = core-avx2) can be used depends on the target architecture that will generate the VectorPairHMM library. The new Java classes are created with “vector logless caching” or “logless caching” algorithms that can improve the VectorPairHMM. Our benchmarking HPC systems are Intel Xeon processors and the vectorization (e.g. SSE 4.1) and advanced vectorization (e.g. AVX, AVX2) are possible to tune the GATK for the architecture aware implementations. The GNU compiler versions ≥4.8 or Intel compiler can be used to support the advanced vectorization instructions. After the above architecture aware GATK source-code compilation, the “libVectorLoglessPairHMM.so” library was generated which can be invoked at Java run-time using “−Djava.library.path = /VectorPairHMM/src/main/c++”. This architecture-aware PairHMM library is used by Java kernel, which can be accelerated the performance of HaplotypeCaller at runtime. We observed upto 180% and 74% improvement in the HaplotypeCaller execution time for the architecture-aware binaries of GATK 3.3 and GATK 3.7 respectively. As a result, the various optimization in the GATK 3.3 brings the improved execution time which is closer to GATK 3.7 results. Further, the optimized HaplotypeCaller GATK 3.7 execution time can be improved upto 124% compared to unoptimized GATK 3.7 pre-compiled jar binary.

We summarized the architecture-specific tuning of HaplotypeCaller which includes (i) the procedure for GATK source code compilation, (ii) architecture specific compiler flags, (iii) configuration options to generate VectorPairHMM library and (iv) example job submission script for architecture aware PairHMM implementation are provided in the Supplemental Data III. Additionally, the generated gVCF files from unoptimized pipeline is compared to optimized pipeline using vcftools^44, 45^. We observed more than 99.993% of same variants listed across the optimized and unoptimized gVCF files. The small variation in the gVCF files are (i) 0.000685145883% of REF mismatches and (ii) 0.007758959268% of ALT mismatches. All the GATK runs are with non-deterministic execution and this is due to multi-threading (−nt or −nct options) and hence the samll variations are acceptable. The summary of vcf-compare results are available in the Supplemental Data III. Furthermore, the performance optimization of (i) Java and garbage collection (ii) CPU frequency scaling and (ii) tuning kernel shared memory parameters are common for any application modules of the GATK pipeline. To understand these performance impact, the complete NGS pipeline using GATK 3.7 version was executed with optimized and unoptimized versions. We observed, the execution time of NGS pipeline reduced by 34.14% using the above changes in the system level optimizations. The complete summary of performance impact is listed in Supplemental data V.

## Conclusion

The execution time of GATK-HaplotypeCaller was reduced using various system level optimizations. We used GATK 3.3 and GATK 3.7 versions of HaplotypeCaller for benchmarking which supports (by default) Java 1.7 and Java 1.8 respectively. The Java 1.8 features are enabled at GATK 3.3 runtime and benchmarked to compare with GATK 3.7. The GATK 3.3 source-code compiled executable are optimized at the system level which includes (i) the parallel garbage collection with heap memory configuration, (ii) the scalability limitation in the GATK-HaplotypeCaller, (iii) the optimal Java I/O temporary directory and (iv) Java 1.8 features for parallel array sorting and bulk data transfer are helped to reduce the execution time by (i) 28%, (ii) 18%, (iii) 24%, and (iv) 34% respectively. These are all some of the features in Java 1.8 and the overall HaplotypeCaller execution time was reduced upto 34%. The advanced vectorization in the PairHMM library and the CPU frequency scaling are common features for GATK 3.3 and GATK 3.7 versions. We observed, 64% and 33% of reduced execution time in GATK 3.3 and 43% and 24% of reduced execution time in GATK 3.7 for PairHMM library and CPU frequency scaling implementations respectively. Moreover, the GATK 3.7 execution time is slightly better (1.7% reduced execution time) than the optimized Java 1.8 implementation of GATK 3.3.

## Electronic supplementary material

Supplemental data I to VI
