# Supplementary material for: Accelerating next generation sequencing data analysis with system level optimizations
Source: Sci Rep. 2017 Aug 22;7:9058. doi: 10.1038/s41598-017-09089-1 (PMC5567265; doi:10.1038/s41598-017-09089-1)
Supplement: Supplementary file 1 — Supplemental data I to VI [file 41598_2017_9089_MOESM1_ESM.doc]

**Supplemental Data**

**Accelerating next generation sequencing data analysis with system level optimizations**

**Nagarajan Kathiresan^*^, Ramzi Temanni, Hakeem Almabrazi, Najeeb Syed, Puthen V Jithesh, Rashid Al-Ali**

Biomedical Informatics Research,

Sidra Medical and Research Center,

Post Box No. 26999,

Doha, Qatar

^*^Corresponding author: **nkathiresan@sidra.org**

**Supplemental Data I:**

**Different type of parallelization in BWA and GATK**

BWA is the most popular genome alignment software. The BWA-MEM algorithm supports long-reads (70bp to 1Mbp) of human genome sequences. To improve the read mapping, we used BWAKIT. Moreover, the BWAKIT package uses the latest version of BWA algorithm called BWA-MEM for alignment, supports HLA typing for high-coverage human data, remap/coordinate sorting of the BAM files and trims the adapter sequence are the various features. This BWAKIT supports thread parallelization by using ‘-t <number of threads>’ option. To understand the scalability and performance improvement, we ran the BWAKIT with -t_1_, -t_2_, ...-t_N_ CPU threads and the GCAT data was used. During the genome alignment, we observed, the shared memory contention and cache/translation look-aside buffer (TLB) miss that affected the poor scalability. We observed, the scalability is linear until CPU threads=8 and poor for CPU threads=32.

Therefore, we partitioned the genome data into independent multiple chunks (but not necessarily equal to the number of CPU threads) based on the level of cache misses happened earlier. The BWAKIT package is executed in a multi-threading mode with every independent chunk of data. The overall execution time is sum of all the execution time of independent chunks of data processing time and this method is referred as “data-parallelization”. For example: the provided GCAT genome data is partitioned into 4 independent chunks of data that provides 0 cache/TLB miss in our system environment. We repeated the BWAKIT execution (our case, 4 times) on every independent chunk with CPU threads=2,4,8 ... N and the execution time will be sum of the all the execution time of independent chunks. Also, the output (partial aligned BAM files) of every BWAKIT execution is merged (in the same order of data partition) into the final alignment file that will become input for the GATK module.

The “data-parallelization” execution time is not impressive comparatively to the thread parallelization. To address these challenges, “data-parallel with concurrent execution” is introduced. In this method, the optimal number of cores is selected based on the scalability limitation using the thread-parallelization concept and the genome data is independently partitioned into multiple chunks based on data-parallelization concept. Then, the BWAKIT is executed on every independent chunk of data concurrently across all the CPUs and optimal number of cores are used for multi-threading within the CPUs. This bring the execution time improvement upto 52% and all these type of parallelization results are summarized in Figure 1.


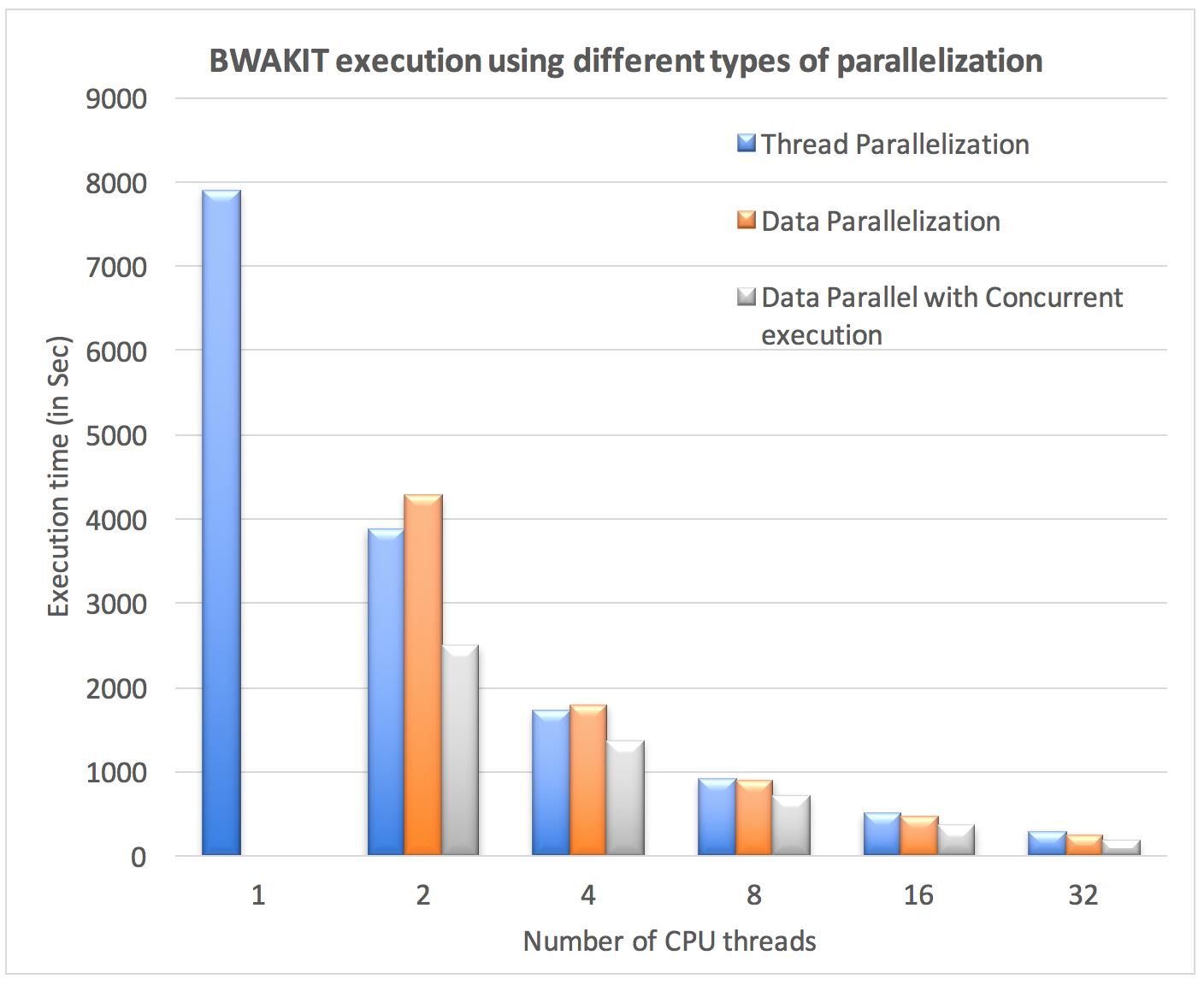


Figure 1. Different types of parallelization in BWAKIT

The GATK-IndelRealigner and GATK-AnalyzeCovariates are used for local realignment of INDELs. These steps are useful for analyzing the BQSR output and it’s not supported by parallelization. Sambamba indexing is used as a standard index for a sorted BAM file. The GATK-RealignerTargetCreator is used for local realignment of BAM files. The Sambamba and GATK-RealignerTargetCreator steps supports thread parallelization. The other GATK modules GATK-BaseRecalibrator, GATK-PrintReads and GATK-HaplotypeCaller are supported by ‘data parallelization’ that are used to generate recalibration-table, manipulate the sequence data and local de-novo assembly respectively. After generating the local de-novo assembly, the GATK-BaseRecalibrator step was repeated to generated recalibration table to compare the confidence level. In GATK, the thread parallelization is controlled by ‘–nt <no. of data threads>’ and the data parallelization is controlled by ‘-nct <no. of CPU threads>’. To understand the best performance parallelization strategies, we tested the Sambamba and GATK with varying number of cores from 1 to N and the summary of scalability shown in Figure 2.


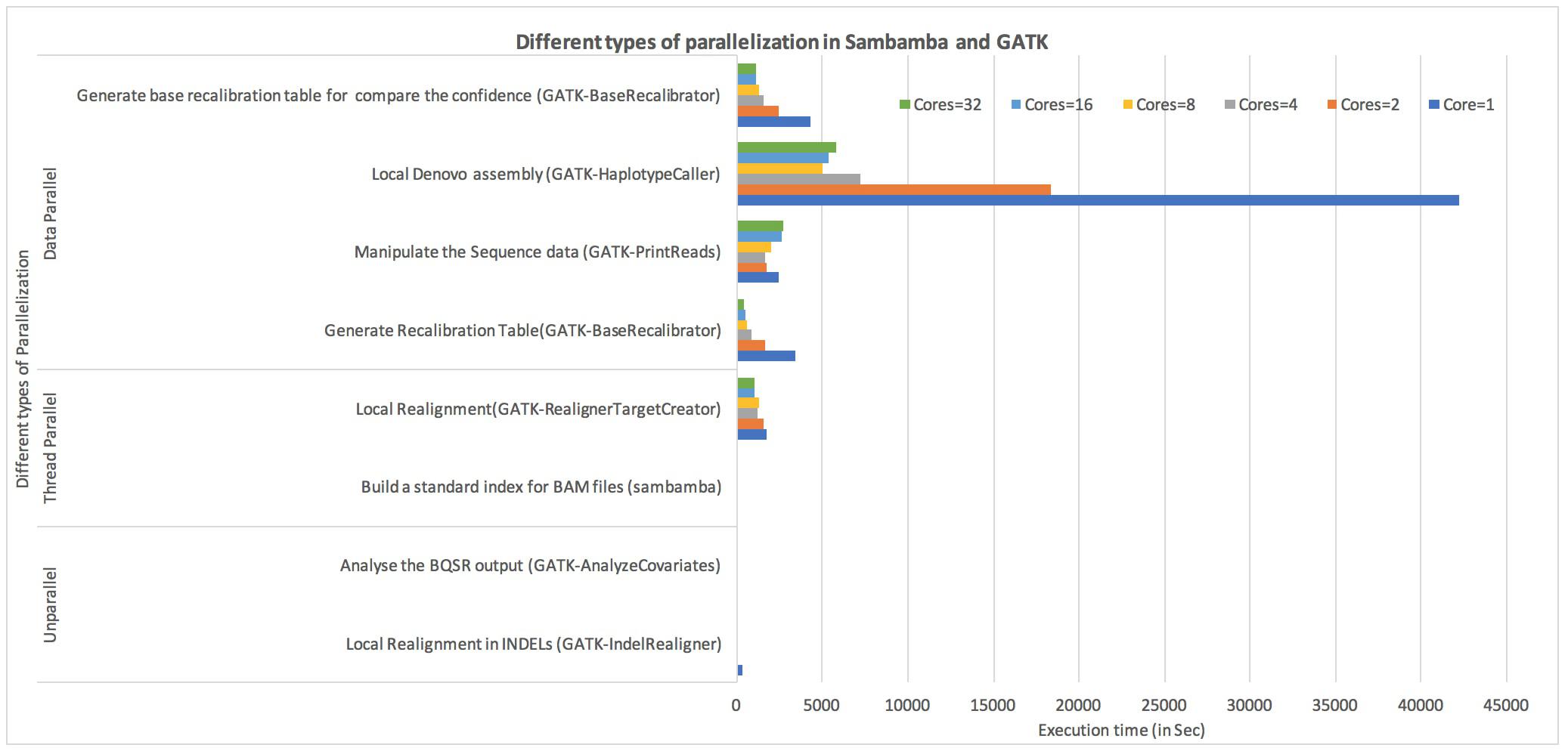


Figure 2. Different types of GATK and Sambamba parallelization

**Supplemental Data II:**

**Benchmarking the different GATK versions**

The GATK software development brings multiple versions (GATK 3.1 to 3.5) over the last two years. Every GATK version includes: new features, advance optimization techniques, additional functionalities and various tools development & enhancements. For our NGS workflow, the functionality or the set of tools which we used (e.g. GATK-Haplotype caller) are common across most of the GATK versions. When the functionality is common, it’s good to compare the execution time across different versions. Accordingly, the optimal GATK version can be selected for the NGS workflow. In general, the execution time of the latest version will be good but it is not necessary due to various factors. To explore this performance difference, we used 3 versions of GATK (version 3.3, 3.4 and 3.5). We benchmarked all these three GATK versions in two flavors: (a) pre-build jar file: download jar file that can be executed in any UNIX based x86_64 system and (b) source-code binary: the download source code was compiled with optimization flags that can take various system architecture features. We benchmarked these 2 flavors of GATK across all these three GATK versions (totaling 6 case studies). To get the consistent results, we used 10 different genome data set from GCAT. The GATK-PrintReads and GATK-HaplotypeCaller steps are taking more than 68% of the total execution time of the NGS workflow. Hence, we benchmarked these workflow steps across different GATK versions. As per the GATK scalability limitation, we used 4 data-parallel threads ( -nct = 4) for GATK-PrintReads and 8 data-parallel threads ( -nct = 8) for GATK-HaplotypeCaller for our benchmarking.

**Observation in GATK-PrintReads**: The GATK pre-build jar file and the source-code binaries gives similar performance of GATK versions 3.4 and 3.3. We observed, GATK 3.5 source-code binary gives more than 23% better performance for most of the data-set and 7% better (average for 10 samples) performance compared to pre-build jar version. When all the source-code binaries of GATK versions are compared to each-other, the GATK 3.5 performance is 18% better than GATK 3.3 and 3.4 and the summary of box plot is shown in Figure 3.


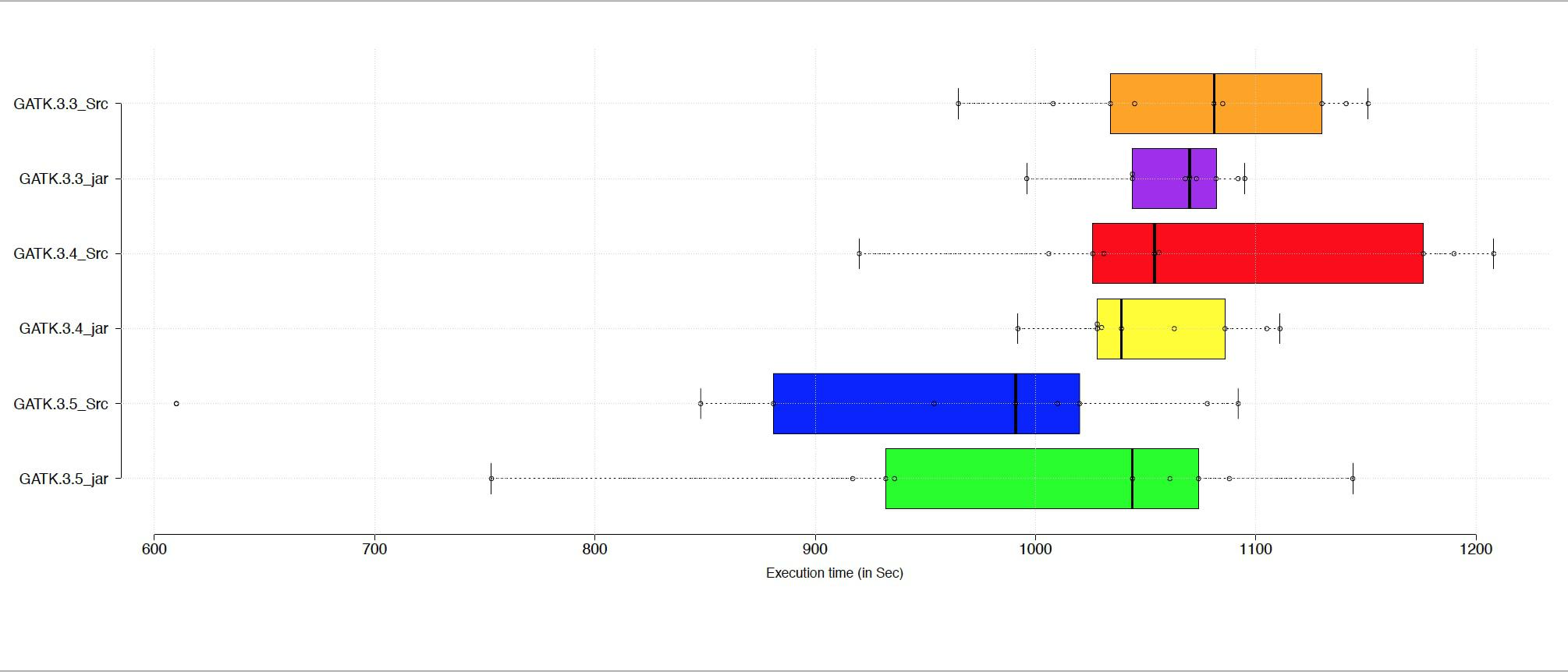


Figure 3. Benchmarking results of different GATK versions using GATK-PrintReads

**Observation in GATK-HaplotypeCaller:** For all the GATK versions of source-code compiled binary gives 12%, 28% and 9% of the improved execution time compared to pre-build jar file of GATK version 3.5, 3.4 and 3.3 respectively. While comparing the GATK source-code binary across all the GATK versions, GATK 3.3 outperform 10% and 8% compared to GATK 3.5 and 3.4 versions respectively. We observed, the GATK 3.4 source-code compiled binary gives 28% better performance than pre-build jar file and the GATK 3.3 source-code compiled binary outperform 10% better performance. The complete box plot summary is shown in Figure 4.


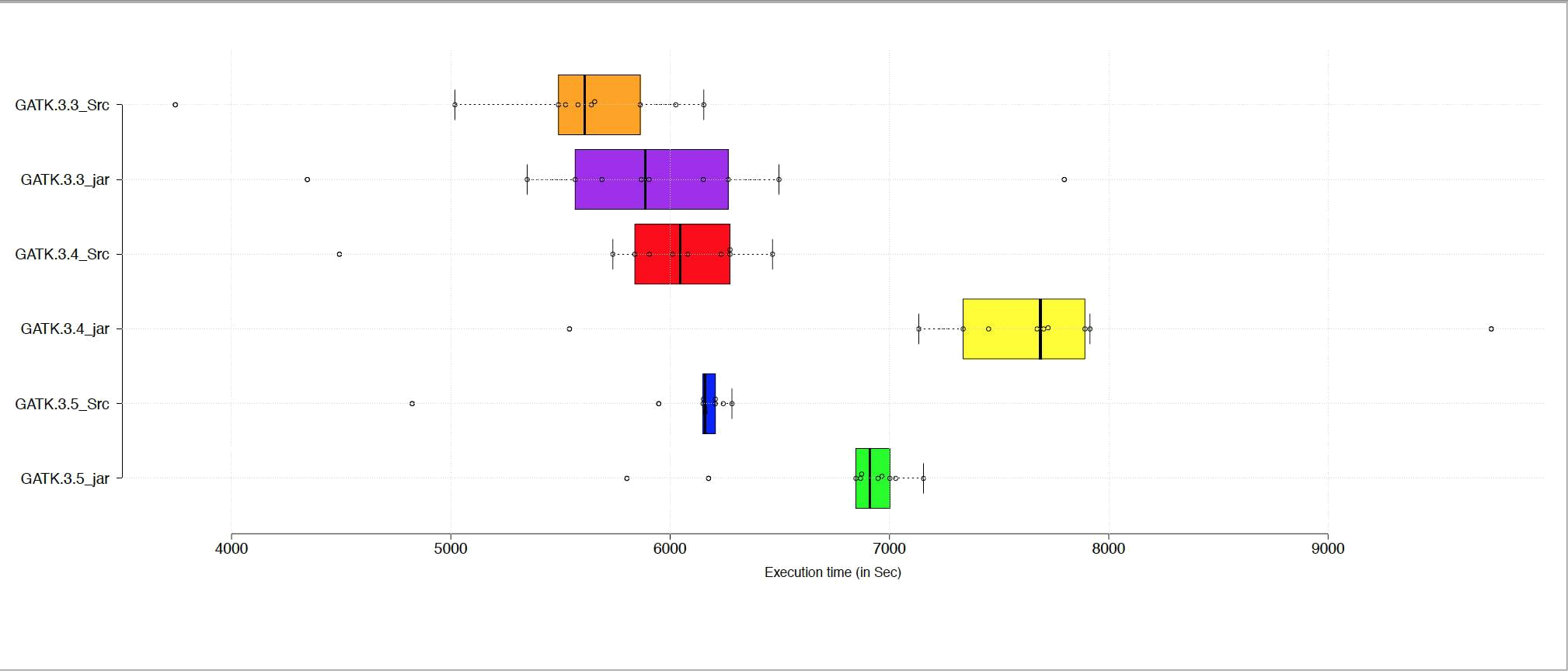


Figure 4. Benchmarking results of different GATK versions using GATK-HaplotypeCaller

As a summary, the GATK 3.5 source-code executable provides 18% better execution time for GATK-PrintReads and the GATK 3.3 source-code executable provides 23% better execution time for GATK-HaplotypeCaller compared to all the GATK versions. As per Amdahl’s law of multi-core optimization, we are selected the GATK 3.3 source-code executable for the NGS workflow because of the time taken in the GATK-HaplotypeCaller is more than other steps.

**Supplemental Data III:**

**Architecture-specific tuning of GATK HaplotypeCaller**

**Pre-Compiled GATK binary:**

The Per-Compiled GATK binary is common for any x86_64 architecture and that can be obtained as follows:

1. Download the GATK pre-compiled binary (E.g. GenomeAnalysisTK-3.5-0-g36282e4.tar.bz2) from broad institute website.
2. Untar the source code ($tar -xjvf GenomeAnalysisTK-3.5-0-g36282e4.tar.bz2)
3. GenomeAnalysisTK.jar file is the GATK pre-compiled binary.
4. To know the GATK version, run the following command:

$ java -jar GenomeAnalysisTK.jar –version

3.5-0-g36282e4

**Source code compilation of GATK:**

**Pre-request:**

Apache Maven and GNU GCC/Intel compiler version 4.8.1 and above.

**Procedure to compile GATK (Example):**

1. Apache Maven is used to compile the GATK jar file. The Linux version of the Apache Maven can be obtained from: <https://maven.apache.org/download.cgi>
2. The downloaded Maven package, get unpacked and the binary path will be set as follows:

**$** wget <http://www-us.apache.org/dist/maven/maven-3/3.5.0/binaries/apache-maven-3.5.0-bin.tar.gz>

**$** tar -xzvf apache-maven-3.5.0-bin.tar.gz

**$** export PATH=/home/naga/apache-maven-3.5.0/bin:$PATH

1. Ensure Java 1.8.0 is available in your compilation environment.

**$** java -version

java version "1.8.0_66"

Java(TM) SE Runtime Environment (build 1.8.0_66-b17)

Java HotSpot(TM) 64-Bit Server VM (build 25.66-b17, mixed mode)

1. Broad Institute github (<https://github.com/broadgsa)> has gatk and gatk-protected repositories. The gatk is for pre-build binary and gatk-protected is for source code (non-commercial purpose) repositories.
2. Clone or download the gatk-protected source code.

**$** git clone <https://github.com/broadgsa/gatk-protected.git> (or)

**$** wget <https://github.com/broadgsa/gatk-protected/archive/3.5.tar.gz>

**$** tar -xzvf 3.5.tar.gz

**$** cd gatk-protected-3.5/

**$** ./ant-bridge.sh

This automated script (./ant-bridge.sh ) will compile the GATK source code.

Equivalent maven command

mvn verify '-Ddisable.shadepackage'

[INFO] Scanning for projects...

[INFO] ------------------------------------------------------------------------

[INFO] Reactor Build Order:

[INFO]

[INFO] GATK Root

[INFO] GATK Aggregator

[INFO] GATK GSALib

[INFO] GATK Utils

…

…

[INFO] ------------------------------------------------------------------------

[INFO] Reactor Summary:

[INFO]

[INFO] GATK Root .......................................... SUCCESS [  0.987 s]

…..

…..

[INFO] External Example ................................... SUCCESS [  1.454 s]

[INFO] GATK Queue ......................................... SUCCESS [ 32.382 s]

[INFO] GATK Queue Extensions Generator .................... SUCCESS [  0.818 s]

[INFO] GATK Queue Extensions Public ....................... SUCCESS [ 31.827 s]

[INFO] GATK Aggregator Public ............................. SUCCESS [  0.005 s]

[INFO] GATK Tools Protected ............................... SUCCESS [ 26.382 s]

[INFO] GATK Package Distribution .......................... SUCCESS [  0.628 s]

[INFO] GATK Queue Extensions Distribution ................. SUCCESS [ 34.403 s]

[INFO] GATK Queue Package Distribution .................... SUCCESS [  0.329 s]

[INFO] GATK Aggregator Protected .......................... SUCCESS [  0.006 s]

[INFO] ------------------------------------------------------------------------

[INFO] BUILD SUCCESS

[INFO] ------------------------------------------------------------------------

[INFO] Total time: 03:17 min

[INFO] Finished at: 2017-05-24T22:37:04+03:00

[INFO] Final Memory: 79M/2590M

[INFO] ------------------------------------------------------------------------

1. The successful compilation will create target/ directory, where the compiled GATK binaries (GenomeAnalysisTK.jar and Queue.jar ) are created.

**$** java -jar target/GenomeAnalysisTK.jar -version

exported

**Procedure to generate VectorPairHMM library:**

1. VectorPairHMM source code is available in gatk-protected-3.5/public/VectorPairHMM directory.
2. To build an architecture specific Java class library, the pom.xml file (line number: 60-62) will be modified as per the compiler availability.
3. To compile this source code, either “mvn install” or “make” is used.

**$** mvn install

[INFO] Scanning for projects...

[INFO]

[INFO] ---------------------------------------------------------------------

[INFO] Building Vectorized PairHMM native libraries 3.5

[INFO] ---------------------------------------------------------------------

[INFO]

…

…

[INFO] ---------------------------------------------------------------------

[INFO] BUILD SUCCESS

[INFO] ---------------------------------------------------------------------

[INFO] Total time: 1.103 s

[INFO] Finished at: 2017-05-24T23:06:28+03:00

[INFO] Final Memory: 26M/1963M

[INFO] ---------------------------------------------------------------------

1. The successful compilation will create target/ directory and the architecture specific java class library (libVectorLoglessPairHMM.so) will be generated.
2. For advanced compilation/architecture aware vectorization (e.g. AVX, AVX2) options, the Makefile in the directory gatk-protected-3.5/public/VectorPairHMM/src/main/c++ will be modified and compiled using the command “make” as follows (Example):

 AVX_FLAGS=-mavx2  -march=corei7-avx

 SSE41_FLAGS=-msse4.1

**$** make

1. The successful compilation will generate libVectorLoglessPairHMM.so library.
2. The example job script to run GATK-HaplotypeCaller with VectorPairHMM library is as follows:

java -Djava.library.path=public/VectorPairHMM/src/main/c++/ \

-XX:+UseParallelGC -XX:ParallelGCThreads=32 \

-jar target/GenomeAnalysisTK.jar \

-T HaplotypeCaller -nct 32 \

--pair_hmm_implementation VECTOR_LOGLESS_CACHING \

-R ref_data/Hsapiens/hs37d5/hs37d5.fa \

-I gcat_set_042.realigned.recal.bam \

--emitRefConfidence GVCF \

--variant_index_type LINEAR \

--variant_index_parameter 128000 \

--dbsnp ref_data/Hsapiens/GRCh37/variation/dbsnp_138.vcf.gz \

-o gcat_set_042.raw.snps.indels.g.32cores.vcf

**Procedure to compare the optimized and un-optimized gVCF files using vcf-tools:**

The example script to run the vcf-tools is as follows:

module load tabix/0.2.4

module load vcftools/0.1.13

bgzip NA12892.PairHMM.raw.snps.indels.g.32cores.vcf

bgzip NA12892.raw.snps.indels.g.vcf

tabix -p vcf NA12892.PairHMM.raw.snps.indels.g.32cores.vcf.gz

tabix -p vcf NA12892.raw.snps.indels.g.vcf.gz

time –p vcf-compare \

NA12892.PairHMM.raw.snps.indels.g.32cores.vcf.gz \ NA12892.raw.snps.indels.g.vcf.gz

The comparison between optimized and un-optimized gVCF results are as follows:

# The command line was: vcf-compare(r953) NA12892.PairHMM.raw.snps.indels.g.32cores.vcf.gz

NA12892.raw.snps.indels.g.vcf.gz

#

#VN 'Venn-Diagram Numbers'. Use `grep ^VN | cut -f 2-` to extract this part.

#VN The columns are:

#VN 1 .. number of sites unique to this particular combination of files

#VN 2- .. combination of files and space-separated number, a fraction of sites in the file

VN 22524 NA12892.PairHMM.raw.snps.indels.g.32cores.vcf.gz (0.0%)

VN 22941 NA12892.raw.snps.indels.g.vcf.gz (0.0%)

VN 100563214 NA12892.raw.snps.indels.g.vcf.gz (100.0%) NA12892.PairHMM.raw.snps.indels.g.32cores.vcf.gz (100.0%)

#SN Summary Numbers. Use `grep ^SN | cut -f 2-` to extract this part.

SN Number of REF matches: 100562525

SN Number of ALT matches: 100554723

SN Number of REF mismatches: 689

SN Number of ALT mismatches: 7802

SN Number of samples in GT comparison: 0

**Supplemental Data IV:**

**Mean/Median coverage of genome data**

The mean and median coverage of the set of genome data are calculated using Picard tools using the following script:

#!/bin/bash

module load java/1.8.0

module load R/R-3.2.2

module load Picard/2.6.0

module load SAMtools/1.3

picard="/gpfs/software/genomics/Picard/2.6.0/build/libs/picard.jar" ;

ref="/gpfs/data_jrnas1/ref_data/Homo_sapiens/hs37d5/Sequences/BWAIndex/hs37d5.fa" ;

javaParams="java -Djava.io.tmpdir=$PWD/tmp/ -Xmx2048m -Xms256m" ;

input=NA12892.aln.bam ;

outname=NA12892 ;

CMD="${javaParams} -jar ${picard} CollectWgsMetrics I=${inputFile} O=${outname}.txt R=${ref}" ;

echo $CMD | time –p

The output of the script will provide mean and median results:

## METRICS CLASS picard.analysis.CollectWgsMetrics$WgsMetrics

GENOME_TERRITORY **MEAN_COVERAGE** SD_COVERAGE **MEDIAN_COVERAGE** … … …

2900340137 **51.613623** 1 5.076832 **53** … … …

**Supplemental Data V:**

**Performance impact of system level optimization in the NGS workflow using GATK 3.7**

| **GATK workflow steps** | Un-optimized GATK 3.7 | Optimized GATK 3.7 | % of performance improvement |
| --- | --- | --- | --- |
|  | (execution time in hours) | |  |
| RealignerTargetCreator | 0.95 | 0.84 | 11.92 |
| IndelRealigner | 10.03 | 8.83 | 12.01 |
| BaseRecalibrator | 4.31 | 3.97 | 7.72 |
| PrintReads | 45.64 | 20.79 | 54.46 |
| HaplotypeCaller | 30.43 | 17.46 | 42.61 |
| BaseRecalibrator2 | 32.68 | 29.80 | 8.80 |
| AnalyzeCovariates | 0.00 | 0.00 | 0.00 |
| **Total Execution time** | **124.04** | **81.69** | **34.14** |

**Supplemental Data VI:**

**BWAKIT pipeline script**

The example BWAKIT run script will be:

~/bwa.kit/**seqtk mergepe** \

~/fastq/gcat_set_038/gcat_set_038_1.fastq.gz \ ~/fastq/gcat_set_038/gcat_set_038_2.fastq.gz \ |

~/bwa.kit/**trimadap** 2> ~/bam-2/gcat_set_038.log.trim \ |

~/bwa.kit/**bwa mem** -p -t32 -R'@RG\tID:gcat_set_038\tLB:gcat_set_038\tSM:gcat_set_038\tPL:ILLUMINA' ~/hs37d5/hs37d5.fa - 2> ~/bam-2/gcat_set_038.log.bwamem \ |

~/bwa.kit/**samblaster** 2> ~/bam-2/gcat_set_038.log.dedup \ |

~/bwa.kit/**samtools sort** -@ 4 -m1G - /bam-2/gcat_set_038.aln;
